# S100A1 as a Potential Diagnostic Biomarker for Assessing Cardiotoxicity and Implications for the Chemotherapy of Certain Cancers

**DOI:** 10.1371/journal.pone.0145418

**Published:** 2015-12-18

**Authors:** Ufuk Eryilmaz, Buket Demirci, Saliha Aksun, Murat Boyacioglu, Cagdas Akgullu, Tevfik Fikret Ilgenli, Hande Sultan Yalinkilinc, Mehmet Bilgen

**Affiliations:** 1 Department of Cardiology, Medical Faculty, Adnan Menderes University, Aydin, Turkey; 2 Department of Medical Pharmacology, Medical Faculty, Adnan Menderes University, Aydin, Turkey; 3 Department of Medical Biochemistry, Medical Faculty, Katip Celebi University, Izmir, Turkey; 4 Department of Pharmacology and Toxicology, Faculty of Veterinary Medicine, Adnan Menderes University, Aydin, Turkey; 5 Department of Cardiology, Konya Selcuklu Private Hospital, Konya, Turkey; 6 Department of Biophysics, Medical Faculty, Adnan Menderes University, Aydin, Turkey; Instituti Ospitalieri di Cremona, ITALY

## Abstract

This study examined the value of blood marker S100A1 in detecting cardiotoxicity induced by chemotherapy agents; trastuzumab and lapatinib, in normal rat heart. The rats were divided into three groups: control (n = 8, no treatment), T (n = 8, one time ip treatment with 10 mg/kg trastuzumab) and L (n = 8, oral treatment with 100 mg/kg/day lapatinib for 7 days). The activities of oxidative stress parameters Malondialdehyde (MDA), Superoxide dismutase (SOD), Catalase (CAT) and Glutathione (GSH) were measured from the extracted cardiac tissues. The levels of troponinI and S100A1 expressions were measured from blood samples. All biomarkers responded to the treatments as they exhibited alterations from their normative values, validating the chemically induced cardiotoxicity. S100A1 expression attenuated significantly (75%), which made the sensitive detection of cardiotoxicity feasible. Assessment of cardiotoxicity with S100A1 may be a valuable alternative in clinical oncology of cancers in some organs such as breast and prostate, as they do not overexpress it to compete against.

## Introduction

S100 proteins are a family of multifunctional proteins characterized by tissue and cell-specific expressions in vertebrates [1]. S100A1 is a member of this family and predominantly expressed in heart, to a lesser degree in skeletal muscle, and at low levels in most normal tissues [2]. S100A1 expression enhances myocardial contractility and is down-regulated following cardiac injury [3]. But, if such behavior would occur in cardiotoxicity has yet to be validated. Current research is focused on identifying reliable biomarkers sensitive and specific to heart failure from different causes [4–10]. If confirmed, S100A1 may become a clinical biomarker for either diagnosis or differentiation in conjunction with other markers, e.g. troponinI and C-reactive protein, in assessing the state of myocardium exposed to toxicity [11–13]. The aim of the current study is to test this potential of S100A1 with experiments performed on rats using chemotherapy agents; trastuzumab and lapatinib, which have proven records of inducing cardiotoxicity [14–19].

## Materials and Methods

The study was approved by the institutional Animal Ethics Committee (Document Number: 4583101/2014/168). Experiments were performed with a total of 24 Wistar rats (12-week-old albino males). The rats were divided into experimental groups. Measurements, indicative of cardiotoxicity, included biochemical parameters sensitive to oxidative stress in myocardium, and blood levels of S100A and troponinI. TroponinI was included as a reference marker [20]. The results were then statistically analyzed and discussed for inferences.

Specifically, the rats were randomly assigned to three groups: control (C, n = 8), trastuzumab (T, n = 8) and lapatinib (L, n = 8) treatments. The control animals were untreated, but the others in groups T and L were administered with the chemotherapy drugs. Trastuzumab (Herceptin^R^, Istanbul, Turkey) was delivered once at a dose of 10 mg/kg/day via intraperitoneal injection on the first day of the study. Lapatinib (Tykerb^®^, GlaxoSmithKline, Istanbul, Turkey) was administered daily at a dose of 100 mg/kg/day by oral gavage for 7 consecutive days. The selected doses were equivalent to those used in the clinics. On day 8, anesthesia was induced by a single intraperitoneal injection of ketamine and xylazine (50 and 5 mg/kg, respectively). The blood samples were collected and the hearts were removed for biochemical analysis.

### Biochemical Analysis

The blood collected from each animal was centrifuged (Hettich Zentrifugen, Mikro 200 R, Tuttlingen, Germany) at 10,000 ×*g* for 10 min at 4°C and the serum was kept at −80°C until the analysis. The serum troponinI level was measured using an immunoassay on Advia Centaur CP (Siemens, Germany) autoanalyzer. The serum S100A1 level was determined by using a rat protein S100A1 ELISA kit (Sensitivity 1.56 ng/ml; Cusabio Biotech, China), according to the manufacturers' instructions. The optical density was measured at 450 nm using an automatic Elisa plate reader (Bioctech, USA).

The activites of Malondialdehyde (MDA), Superoxide dismutase (SOD), Catalase (CAT) and Glutathione (GSH) were measured by following the procedures, as in our previous studies[21–23]. The dissected heart tissue from each animal was homogenized at 2000 rpm/min (1/10 w/v) using a Teflon-glass stirrer (IKA Overhead Stirrer, Germany) in 150 mM phosphate buffer (pH 7.4) on ice. The homogenate was centrifuged (Hettich Zentrifugen, Mikro 200 R, Tuttlingen, Germany) at 6000×g for 10 min at 4°C. The supernatants were frozen at −80°C (Glacier Ultralow Temperature Freezer, Japan) until the analysis. The protein content in supernatants were determined by a spectrophotometer (Shimadzu UV-1601, Kyoto, Japan) using commercially available kits for the Biuret method (Archem Diagnostic Ind. Ltd., Istanbul, Turkey). The MDA concentrations were measured at 532 nm according to the method of Ohkawa et al. [24]. Lipid peroxidation of the tissue homogenate was determined by the formation of thiobarbutric acid reactive substances. Tissue MDA concentration expressed as nmol/mg tissue protein (absorbance coefficient ε = 1.56x105/M/cm). SOD activity was determined according to the method of Sun et al. [25], and the absorbance was measured at 560 nm. This method is based on the inhibition of nitro blue tetrazolium reduction using the xanthine: xanthineoxidase system as a superoxide generator. The measured value indicated the degree of inhibition of this reaction. The results were shown as U/mg of tissue protein. CAT activity was determined by measuring the decomposition of hydrogen peroxide at 240 nm, and expressed as k/mg of tissue protein, where k is the first-order rate constant [26]. Total GSH level in supernatants were determined according to the method of Tietze [27]. The supernatant was precipitated and measured with a kinetic assay using 5,5′-dithiobis (2-nitrobenzoic acid). Absorbance was measured at 412 nm. The measured GSH concentration was compared with GSH aqueous standard solution (Sigma Chemical Co., St. Louis, Missouri, USA) and expressed as mg/g tissue protein. All these enzymatic activity assays were analyzed in duplicate, and the readings were averaged to reach a final value for each assay.

### Statistical Analysis

All measurements for the biomarkers (MDA, SOD, CAT, GSH, troponinI and S100A1) were tabulated for the experimental groups (C, T and L) and analyzed statistically using nonparametric tests by assuming non-Gaussian distribution due to low group size (n = 8). Statistically significant differences in the measurements were determined by using multiple group comparison with both Mann-Whitney U test as applied between two groups (T or L with C**)** and Kruskal Wallis test as applied to all three groups. Descriptive statistics of each marker were represented with box plots (minimum, 25% percentile, median, 75% Percentile, maximum) and in a table (mean ± standard error) values. Two tailed *P* values were obtained from the statistical tests and reported explicitly. The level of statistical significance was set at *P <* 0.05.

## Results

**Fig 1:**
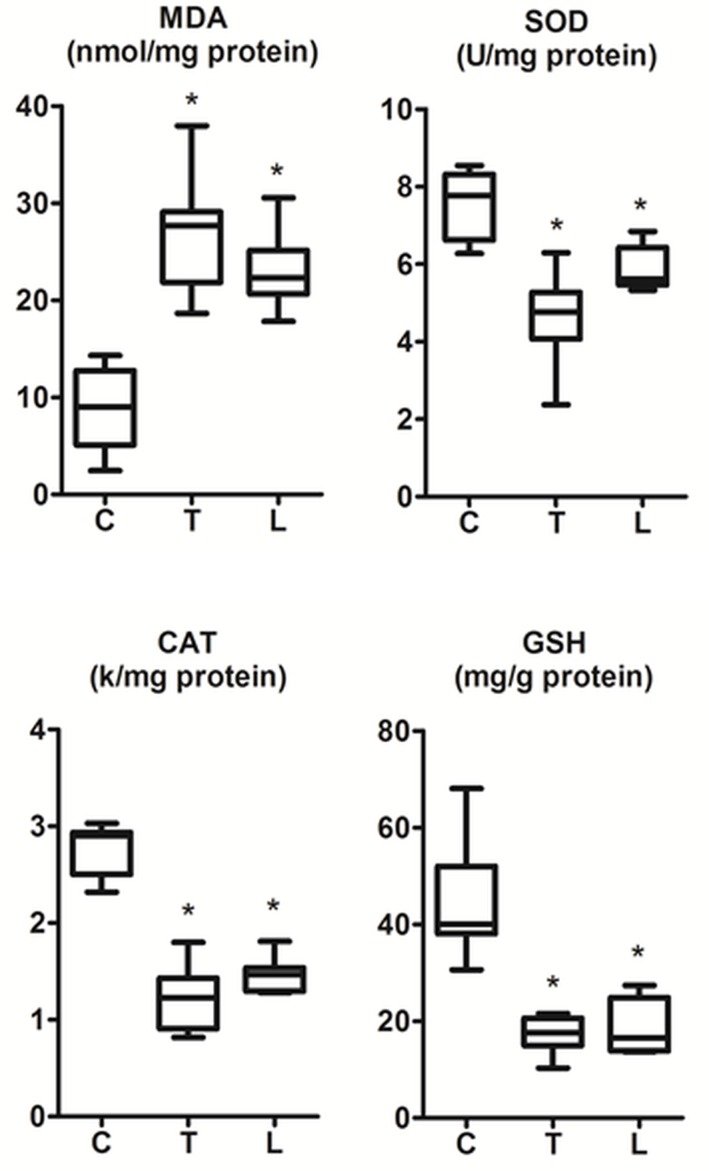
Oxidative stress parameters as measured from the cardiac tissues extracted from the animals in all experimental groups (each with n = 8). Panels show graphs for the biochemical markers Malonyldialdehyde (MDA); Superoxide dismutase (SOD); Catalase (CAT); Glutathione (GSH). Multi group Kruskal Wallis test yielded *P* = 0.0003, 0.0003, 0.0002 and 0.0005, respectively. * indicates statistically significant difference against the control group C (see Table 1).

**Fig 2:**
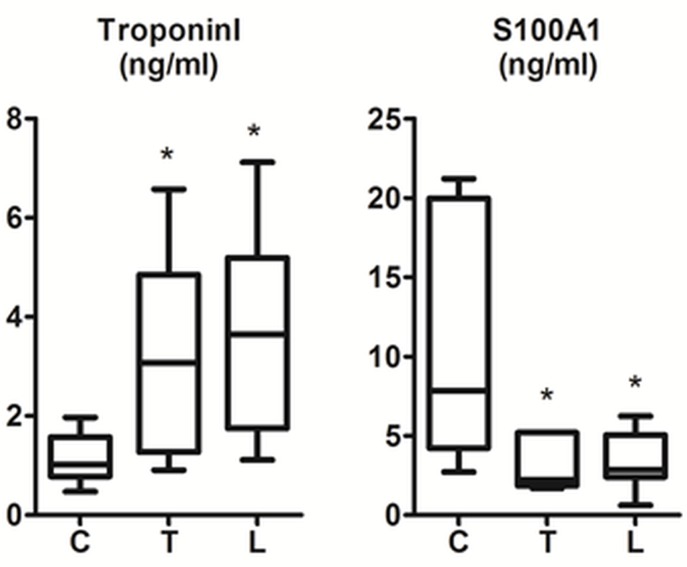
Levels of cardiac injury parameters as measured from the bloods collected from all experimental groups (each with n = 8). Panels show graphs for the markers TroponinI and S100A1. Multi group Kruskal Wallis test yielded *P* = 0.0280 and 0.0354, respectively. * indicates statistically significant difference against the control group C (see Table 1).

The rats in all groups tolerated the procedures and survived seven days without visible side effects or complications. Measurements of parameters from all groups were summarized with plots in Fig 1 and Fig 2, and Table 1. Readings from the control group were within the normal range as reported in the literature [21–23]. But, those in the trastuzumab and lapatinib treatment groups indicated substantial changes in the biochemistry of the hearts (Fig 1 and Table 1). The level of MDA increased significantly, but the activities of SOD, CAT and GSH attenuated considerably. These results confirmed that exposure to the chemotherapy treatment drugs; trastuzumab and lapatinib, indeed induced toxic injury through oxidative damage in the rat hearts.

**Table 1:** Antioxidant and oxidant status in the heart tissues and serum markers obtained from the experimental groups.

Groups	Biomarkers					
	MDA (nmol/mg)	SOD (U/mg)	CAT (k/mg)	GSH (mg/g)	TroponinI (ng/ml)	S100A1 (ng/ml)
**Control** (C)	8.86 ±1.46	7.59 ±0.30	2.76 ±0.09	44.46 ±4.12	1.14 ±0.21	10.65 ±3.19
**Trastuzumab** (T)(10 mg/kg)	27.11 ±2.10*	4.62 ±0.40*	1.22 ±0.11*	17.33 ±1.30*	3.22±0.88*	3.08 ±0.68*
**Lapatinib** (L)(100 mg/kg/day)	22.96 ±1.34*	5.88 ±0.19*	1.46 ±0.06*	18.70 ±1.96*	3.68 ±0.79*	3.37 ±0.70*
*P*_C-T_	0.0002	0.0003	0.0009	0.0002	0.0411	0.0260
*P*_C-L_	0.0002	0.0019	0.0007	0.0004	0.0140	0.0350

MDA, Malondialdehyde; SOD, Superoxide dismutase; CAT, Catalase; GSH, Glutathione.

The readings were expressed as (mean ± standard error).

The *P* values at the bottom two rows were obtained from Mann-Whitney U test performed between the measurements from the two groups (C and T or C and L).

* indicates statistically significant difference against the group C.

TroponinI and S100A1 were detectable in the serums acquired from all groups, but their expressions responded oppositely to cardiotoxicity, as seen in Fig 2 and Table 1. The effect was positive on the troponinI, but negative for S100A1 level.

## Discussion

Trastuzumab and lapatinib are clinically approved drugs used for treating tumors in human patients, but they also induce side effect of toxicity in organs including heart [28]. Exposure to these chemotherapy agents results in excess production of oxygen free radicals and reduction of antioxidant enzyme activities in vital organs. The generation of free radicals constitutes one of the underlying mechanisms for the intoxication of heart [18]. In preclinical stages, the resulting tissue damage and apoptosis is typically identified by ex vivo biochemical analysis involving the well-known indicators (MDA, SOD, CAT, GSH). In the current study, the changes in the activities of these markers indicated that trastuzumab and lapatinib at the administered doses induce severe oxidative damage in the cardiac tissue of rat. These findings supported the previously published reports [15, 20].

Blood based biomarkers are important in clinical practice as they can be detected by assays, which are easy to perform and produce tissue specific and reproducible quantitative data [17]. The current investigation indicated that the cardiac tissue in response to intoxication altered the expressions of both troponinI and S100A1. TroponinI is an established serum biomarker, which correlates with the damage in cardiomyocytes [29–32]. The amplification of troponinI was not a surprise, and in this study, this feature served as a reference confirmatory proof for depicting the presence of cardiac injury. In clinics, the elevation of troponinI is considered as a common sign of cardiotoxicity in patients going through chemotherapy [15, 30, 31, 33, 34]. But, the degree of cardiac dysfunction in these patients is typically evaluated using sensitive radiological tools [35].

In myocardial injury following an infarct or cocaine-use or neurological dysfunction, S100A1 expression is known to be down-regulated [36]. But, prior to this current investigation, it was not clear if such response would also occur for chemically induced cardic injury. The results from this study showed the feasibility of detecting cardiotoxicity with the blood-drived marker S100A1. This outcome has important implications from the perspective of clinical practice, as explained below.

## Conclusion and Future Work

Treatment with trastuzumab and lapatinib induces oxidative damage in myocardial tissue. The resulting cardiotoxicity is detectable with the attenuation in the S100A1 expression or increase in the troponinI level in blood.

In many tumors, the expressions of S100 proteins are altered [37]. However, S100A1 is up-regulated only in cancers of kidneys, skin and ovary. Thus, S100A1 screening following the chemotherapy of these specific cancers may not accurately reveal cardiotoxicity. However, reliably assessing cardiotoxicity in treating the cancers of other organs such as breast and prostate may still be a possibility and warrants further exploration [38]. This study establishes the very first step towards this direction by demonstrating the S100A1 response to cardiotoxicity induced by trastuzumab and lapatinib in otherwise normal rats.

In experimental investigations with S100A1 transgenic and knockout mice, down-regulation of S100A1 protein was shown to contribute to contractile dysfunction after myocardial infarction [39]. In myocardium of knockout mice, severely impaired Ca^2+^cycling and β adrenergic signaling were present in sarcoplasmic reticulum. Also, mitochondrial dysfunction was involved in stress-induced cardiomyopathy [40]. Thus, following chemotherapy treatment, similar pathophysiological mechanism may be responsible for the loss of contractile performance and propensity toward the heart failure. Investigation of this potential aspect with transgenic and knockout models also remains for the future.
